# Neuroimmune system-mediated renal protection mechanisms

**DOI:** 10.1007/s10157-021-02062-3

**Published:** 2021-04-20

**Authors:** Tsuyoshi Inoue

**Affiliations:** Department of Physiology of Visceral Function and Body Fluid, Nagasaki University Graduate School of Biomedical Sciences, 1-12-4, Sakamoto, Nagasaki, 852-8523 Japan

**Keywords:** Vagus nerve stimulation, Acetylcholine receptor, Cholinergic anti-inflammatory pathway, Optogenetics, Acute kidney injury, Nervous system

## Abstract

The autonomic nervous system plays an important role in maintaining homeostasis in organisms. Recent studies have shown that it also controls inflammation by directly altering the function of the immune system. The cholinergic anti-inflammatory pathway (CAP) is one of the neural circuits operating through the vagus nerve. Acetylcholine released from the terminal of the vagus nerve, which is a parasympathetic nerve, acts on the α7 nicotinic acetylcholine receptor of macrophages and reduces inflammation in the body. Previous animal studies demonstrated that vagus nerve stimulation reduced renal ischemia–reperfusion injury. Furthermore, restraint stress and pulsed ultrasound had similar protective effects against kidney injury, which were mainly thought to be mediated by the CAP. Using optogenetics, which can stimulate specific nerves, it was also revealed that activation of the CAP by restraint stress was mediated by C1 neurons in the medulla oblongata. Nevertheless, there still remain many unclear points regarding the role of the nervous and immune systems in controlling renal diseases, and further research is needed.

## Introduction

It has long been suggested that the immune system depends on the state of mind, as validated by the phrase "disease is from the mind." So far, it has been revealed that immune cells, such as macrophages, dendritic cells, T cells, and B cells, express receptors for neurotransmitters on their surface and directly receive signals from the nervous system to change their dynamics [1]. Recently, it was reported that inflammatory diseases, including kidney disease, involve such control of the immune system by the autonomic nervous system [2, 3]. This observation has progressed to therapeutic applications in some diseases. This paper outlines the relationship between the nervous system and the immune system, with a focus on kidney disease.

## Neuron–immune interactions and the cholinergic anti-inflammatory pathway (CAP)

The hypothalamic–pituitary–adrenal axis is a glucocorticoid-mediated innate immune response mechanism that is controlled by the central nervous system, specifically the paraventricular nucleus of the hypothalamus [4]. It was discovered in the 1920s that the nervous system regulates inflammatory responses via hormones. Subsequently, attention was focused on neural circuits mediated by inflammatory cytokines, such as tumor necrosis factor (TNF)-α and interleukin-1, and receptors, and it was found that the vagus nerve was involved in this reaction [5, 6]. Cytokines produced by damaged cells activate afferent sensory nerves and bind to the receptors on afferent vagus nerves to signal solitary nuclei of the brain stem and regulate the immune response of peripheral tissues. However, because these anti-inflammatory effects are also linked to the hypothalamic–pituitary–adrenal axis, it was unclear how the vagal afferents were activated.

A breakthrough in the hypothesized interaction between the nervous and immune systems was the proposal of the inflammatory reflex by Tracey's group [7]. In 2000, it was discovered in a rat sepsis model created by lipopolysaccharide administration that direct electrical stimulation of the vagal efferent tract suppressed the production of TNF-α (an inflammatory cytokine) and, consequently, shock. This effect was obviated by vagotomy, indicating that it involved a series of reflex reactions by neural circuits [8]. At the same time, it was identified that the reaction was stimulated by acetylcholine (ACh) through its receptors on macrophages. They named this series of vagus nerve-mediated responses as the CAP. Later, using α7 nicotinic acetylcholine receptor (α7nAChR)-knockout mice, it was clarified that the α7 subunit of the nicotinic receptor was important among ACh receptors [9]. In experiments using a sepsis mouse model, it was found that the spleen contributed to the production of TNF-α, and splenectomy diminished the effect of vagus nerve stimulation (VNS). This revealed the importance of the spleen in the CAP [10].

**Fig. 1:**
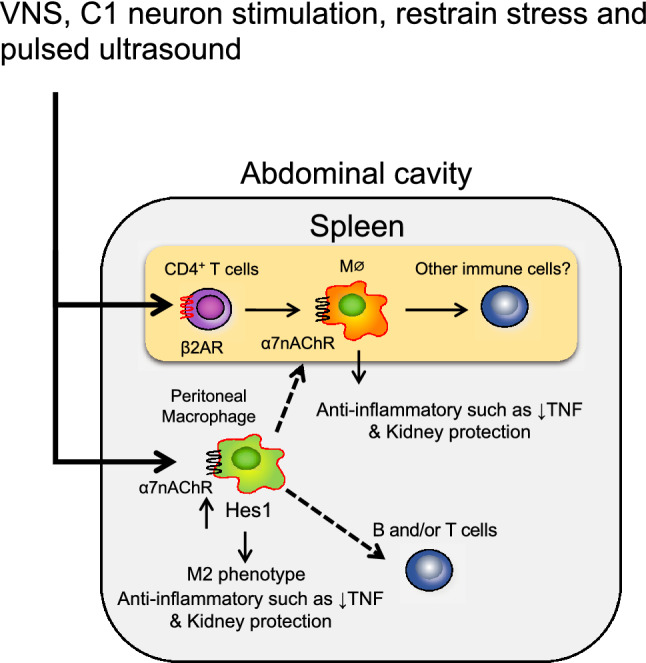
Current understanding of the cholinergic anti-inflammatory pathway in kidney injury. Based on reports centered on sepsis and our data on the kidney, the cholinergic anti-inflammatory pathway in kidney diseases, as understood to date, is shown. It is assumed that various immune cells work cooperatively in the pathway to protect the kidney. β2AR, β2 adrenergic receptor, α7nAChR, α7 nicotinic acetylcholine receptor

ACh is released from vagus nerve endings, whereas the splenic nerve is adrenergic, suggesting the involvement of other intervening factors between the vagus and splenic nerves. Although there are still unclear points about its comprehensive processes, the current understanding of the CAP in kidney injury is based on the findings detailed and illustrated in Fig. 1. Local inflammation is sensed by the afferent vagus nerve and transmitted to the central nervous system. After that, it is transmitted to the spleen via the efferent vagus and splenic nerves, and noradrenaline is released from the splenic nerve endings. Noradrenaline binds to β2 adrenergic receptors present on specific T cells (CD4 + CD44^high^CD62L^low^ memory T cells) that have choline acetyltransferase (ChAT), an ACh synthase, and releases ACh [11]. It is believed that the ACh released from CD4 + T cells binds to α7nAChR on macrophages and attenuates the release of inflammatory cytokines, such as TNF-α, from macrophages, thus suppressing inflammation. However, it is still unknown how the parasympathetic vagus nerve activates the sympathetic splenic nerve. As mentioned earlier, the CAP may involve more complex mechanisms.

## VNS and acute kidney injury (AKI)

**Fig. 2:**
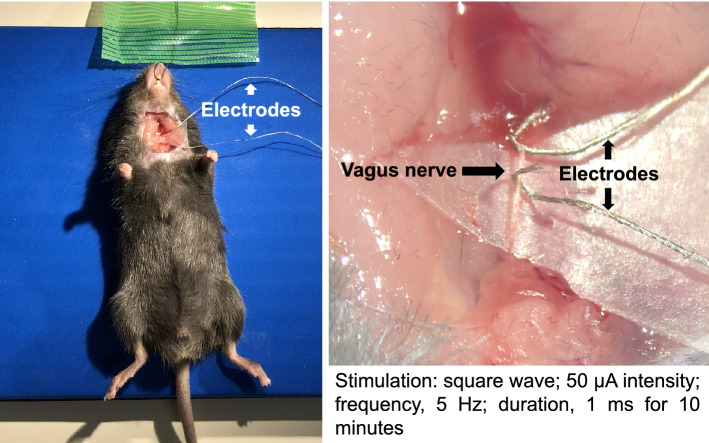
Vagus nerve stimulation. The method of vagus nerve stimulation by electricity is shown. After identifying the vagus nerve in the left neck, two electrodes were applied to the nerve to perform electrical stimulation

**Fig. 3:**
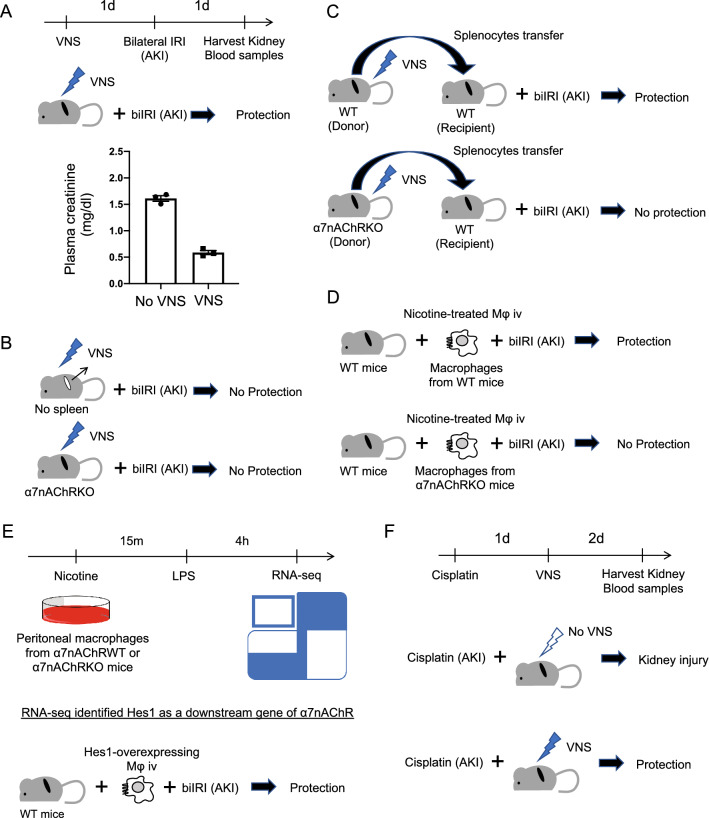
Summary of the role of vagus nerve stimulation (VNS) on acute kidney injury. **a** Prior VNS protects the kidney from ischemia–reperfusion injury. **b** The kidney protective effect by VNS is lost in splenectomized or α7nAChR-deficient mice. **c** Adoptive transfer of splenocytes from mice who have α7nAChR and received VNS protects the kidney. **d** Adoptive transfer of nicotine-treated macrophages isolated from wild-type (WT) mice protect the kidney. The protective effect is lost when nicotine-treated macrophages isolated from α7nAChR-deficient mice are adoptively transferred. **e** RNA-seq identified Hes1 as a downstream gene of α7nAChR in macrophages. Nicotine was used as an agonist for acetylcholine receptors and lipopolysaccharide was used to induce inflammation in macrophages. **f** VNS has an organ-protecting effect even after the induction of kidney damage by cisplatin

AKI is a rapid deterioration of renal function caused by various factors such as ischemia, sepsis, and drugs. Both innate and adaptive immunity are involved in the pathology of AKI, and various immune cells are intricately intertwined in space and time to cause inflammation [12]. It is not known how the neuroimmune linkage affects this condition. Therefore, we first investigated how VNS affected the pathophysiology of AKI [13]. The most commonly used model of AKI is bilateral ischemia–reperfusion injury (IRI), which clinically corresponds to renal injury after cardiac surgery or transplantation. Our method of stimulation of the vagus nerve with electricity is shown in Fig. 2. When VNS was performed on wild-type mice 24 h before bilateral IRI, renal function and renal pathological findings were significantly improved (Fig. 3a), and systemic TNF-α level was also decreased [13]. However, this anti-inflammatory effect was not observed in splenectomized or α7nAChR-knockout mice, suggesting that both the spleen and α7nAChR are involved in VNS-induced kidney protection (Fig. 3b) [13]. Furthermore, when bilateral IRI was induced after splenocytes were adoptively transferred from wild-type mice that received VNS, renal damage of the recipient was significantly reduced. In contrast, similar experiments conducted with α7nAChR-knockout mice as splenocyte donors showed no effect, demonstrating that the anti-inflammatory effect of VNS on IRI is mediated by α7nAChR-positive splenocytes (Fig. 3c) [13].

What exactly does VNS change in splenocytes? Results of previous experiments showed that the phenotypes of macrophages migrating into the kidney after injury were changed by VNS [13]. The levels of arginase 1, a well-known marker of anti-inflammatory macrophages (M2), were increased significantly in the kidneys of wild-type mice that underwent IRI after VNS. Increased expression of arginase 1 was not observed in α7nAChR-knockout mice treated with VNS. It is believed that such macrophage phenotypic changes can explain at least part of the anti-inflammatory and organ-protective effects in the kidney elicited by VNS.

From these results, we focused on α7nAChR-positive macrophages and explored their role in the CAP [14]. When peritoneal macrophages from wild-type mice were pretreated with nicotine (an agonist of nicotinic receptors including α7nAChR) and transplanted into another mouse, they acted protectively against the recipient's kidney IRI. This effect was not observed in experiments using α7nAChR-knockout mice as donors, suggesting that α7nAChR on macrophages is important (Fig. 3d). Therefore, among the genes whose expression increased significantly when peritoneal macrophages collected from wild-type mice were treated with nicotine, the genes whose expression did not increase when peritoneal macrophages of α7nAChR-knockout mice were treated with nicotine were analyzed using RNA-seq. Upon screening, one of the basic helix–loop–helices, hairy and enhancer of split-1 (Hes1), was extracted as a candidate downstream gene. VNS induced Hes1 expression in peritoneal macrophages, and knocking down Hes1 in peritoneal macrophages suppressed the anti-inflammatory effect observed with nicotine administration. In addition, overexpression of Hes1 showed an anti-inflammatory effect, and the macrophage phenotype changed toward M2. Furthermore, adoptive transfer of Hes1-overexpressing macrophages into mice reduced renal IRI. These results suggest that VNS increased the expression of Hes1 through α7nAChR of macrophages and exerted a renal protective effect by changing the macrophage phenotype toward M2 (Fig. 3e).

In the above studies, we performed VNS before inducing kidney damage, and thus concluded that it had a renal protective effect. However, most clinical settings present patients with pre-existing kidney injury; therefore, we focused on the renal protective effect of VNS after the kidney was damaged. Cisplatin is a widely used anticancer agent. Approximately one-third of the patients who receive cisplatin suffer from AKI. Therefore, we evaluated the effect of VNS using an animal model of cisplatin-induced nephropathy. The results confirmed that VNS had an organ-protective effect, even post-cisplatin-induced nephropathy, and that CCL2 and CCL11, factors related to immune cell chemotaxis, were suppressed in macrophages through α7nAChR (Fig. 3f) [15].

## C1 neuron stimulation activates the CAP and protects the kidney

With the advent of optogenetics, it is now possible to manipulate the activity of specific neurons with light [11] and grasp the roles of nerves more accurately. C1 neurons in the medulla oblongata project into the intermediolateral cell column and the dorsal motor nucleus of the vagus and control the sympathetic and vagus nerves. C1 neurons are activated by inflammatory cytokines and lipopolysaccharide, and it has been thought that they are deeply involved in the inflammatory process; however, the details remain unknown. Therefore, we attempted to elucidate the role of C1 neurons using an AKI model (bilateral IRI). When short-term restraint stress (restraining mice in a closed position thereby activating autonomic nerves) was applied, renal damage after bilateral IRI was markedly suppressed. This anti-inflammatory effect after restraint stress was not observed in α7nAChR-knockout mice. Furthermore, when spleen cells collected from a mouse subjected to restraint stress were transplanted into another mouse and IRI was then performed on the recipient, renal damage was also alleviated. In other words, α7nAChR and spleen cells were both important, suggesting that the CAP is also involved in the renal protective effect of restraint stress.

**Fig. 4:**
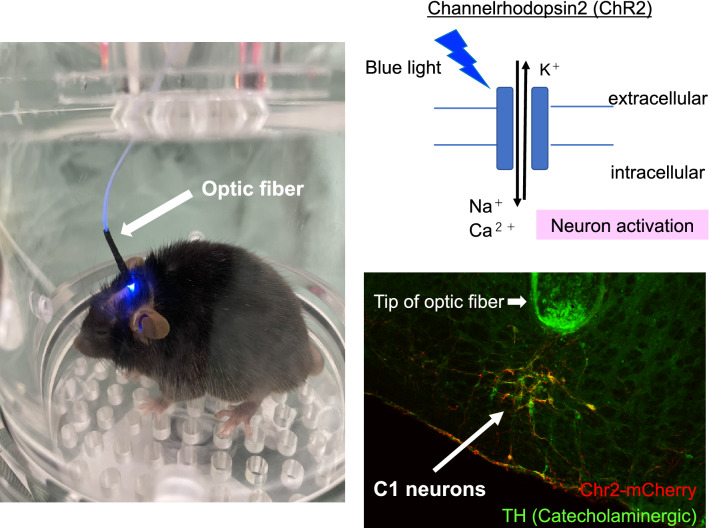
C1 neuron stimulation with optogenetics. Channelrhodopsin-2 (ChR2) is a photoactivated non-selective cation channel, and neurons expressing ChR2 can be activated by blue light. Five to six weeks after virus injection with ChR2 into C1 regions and implantation of the optic fiber, C1 neurons express ChR2 and can be stimulated by a blue laser. ChR2 expression was confirmed in the C1 neurons (catecholaminergic) in the medulla oblongata. A small lesion surrounded by green autofluorescence shows the location of the tip of the optical fiber

We tested the hypothesis that C1 neurons were involved in the activation of the CAP. Channelrhodopsin-2 (a photoactivated non-selective cation channel found in green algae) [16] was used with optogenetics to selectively stimulate C1 neurons. This stimulation reduced renal damage after bilateral IRI as well as restraint stress (Fig. 4) [17]. When restraint stress was applied after selectively inhibiting C1 neurons, this renal protective effect disappeared, demonstrating that the renal protective effect of restraint stress was mediated by C1 neurons. However, the renal protective effect of C1 neuron stimulation was not diminished by blockade of the parasympathetic nervous system by subdiaphragmatic vagotomy and administration of an inhibitor of the corticosterone receptor, indicating that C1 neuron stimulation-induced kidney protection was mediated by the sympathetic nervous system. This suggests that the C1 neuron-related kidney protection mechanism is different from VNS, which is considered to be the parasympathetic nervous system, although the general framework of the CAP is common for both.

## Pulsed ultrasound activates the CAP

**Fig. 5:**
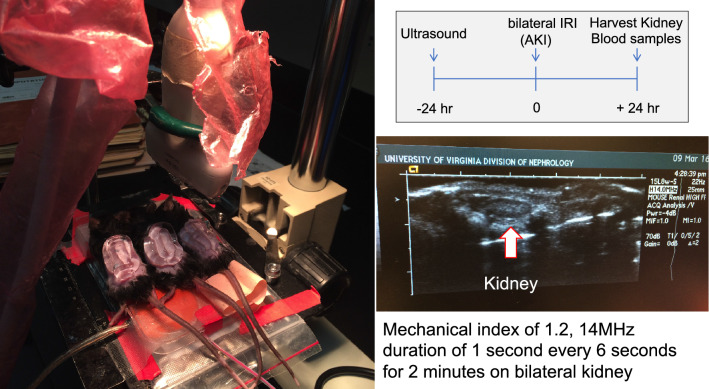
Pulsed ultrasound treatment for the prevention of acute kidney injury. This figure shows how we treated mice with pulsed ultrasound (left). The fur was shaved and then removed completely using a depilatory. Mice were then placed on a modified microscope stage that was positioned under an ultrasound transducer held in place with a ring clamp. A pre-warmed ultrasound gel was then placed on the depilated skin for ultrasound application. Mouse body temperature was monitored using a rectal probe (Fine Science Tools, Foster City, CA, USA) and maintained at 36 ± 0.5 °C with a heating pad and heat lamp. The white arrow shows the kidney. The actual pulsed ultrasound stimulation was performed targeting the range shown in the photograph (right)

We have also shown the preventive effect of pulsed ultrasound stimulation on AKI [14, 18, 19]. An ultrasound device (Sequoia 512) that is used in actual clinical practice (15L8w probe, Acuson, Indianapolis, IN, USA) was used for this study. Pulsed ultrasound stimulation was performed under the contrast imaging mode for 2 min at 7 MHz, and a mechanical index of 1.2. Renal damage was prevented by this ultrasound stimulation of the abdomen 24 h before bilateral renal IRI (the effect weakened with time but continued until 5 days) (Fig. 5). In addition, this effect was lost when the spleen was removed in advance, suggesting that the spleen is important for the renal protective effect of ultrasound. When Rag1-knockout mice lacking T and B cells received pulsed ultrasound stimulation and renal IRI, the protective effect was lost. Introducing wild type CD4-positive T cells into Rag1-knockout mice restored the protective effect, suggesting that CD4-positive cells are involved in the renal protection mechanism by ultrasound stimulation. In addition, this protective effect disappeared when splenectomy was performed before the transfer of CD4-positive cells [18]. Furthermore, blocking splenic sympathetic nerves with 6-hydroxydopamine, a neurotoxin that selectively destroys catecholamine-producing nerve endings, exacerbated renal IRI [19]. Collectively, these results suggest that the spleen, splenic sympathetic nerves, and CD4-positive T cells are all involved in the renal protective effect of pulsed ultrasound. Furthermore, the use of α7nAChR inhibitors eliminated the protective effect of ultrasound [18].

Levels of cytokines, such as TNF-α, were decreased in plasma and kidneys of mice in which the renal protective effect was induced by prior ultrasound stimulation. The renal protective effect of ultrasound has been confirmed in a cecal ligation and puncture sepsis model [19] and in a renal IRI experiment using pigs (unpublished data). From the above results, it was considered that the preventive effect of ultrasound on AKI was mediated by the CAP as well as VNS, restraint stress, and C1 stimulation. Recently, Aibara et al. also showed that pulsed ultrasound ameliorated inflammation and renal fibrosis in hypertensive and diabetic nephropathy [20]. Thus, pulsed ultrasound treatment is a non-invasive therapeutic tool with no side effects, and this modality could be very useful for protecting the kidney. However, as shown in Fig. 5, the ultrasound probe used in our studies is considerably larger than the body of a mouse, and it is still unknown how and which organ the ultrasonic waves actually stimulate. There are some reports of how ultrasonic waves stimulate or inhibit nerves and cells. Thermal effects [21, 22] and mechanical effects [23, 24] are suggested as the mechanisms of nerve stimulation or inhibition by ultrasonic waves using ex vivo or in vitro studies. Recently, one paper demonstrated that focused ultrasound can elicit peripheral nerve stimulation with a physiological response in vivo [25]. Thus, it is possible that abdominal ultrasonography stimulates afferent nerves and ganglia in the abdominal cavity, and it is necessary to elucidate the detailed CAP activation mechanism in the future.

## Kidney nervous system

**Fig. 6:**
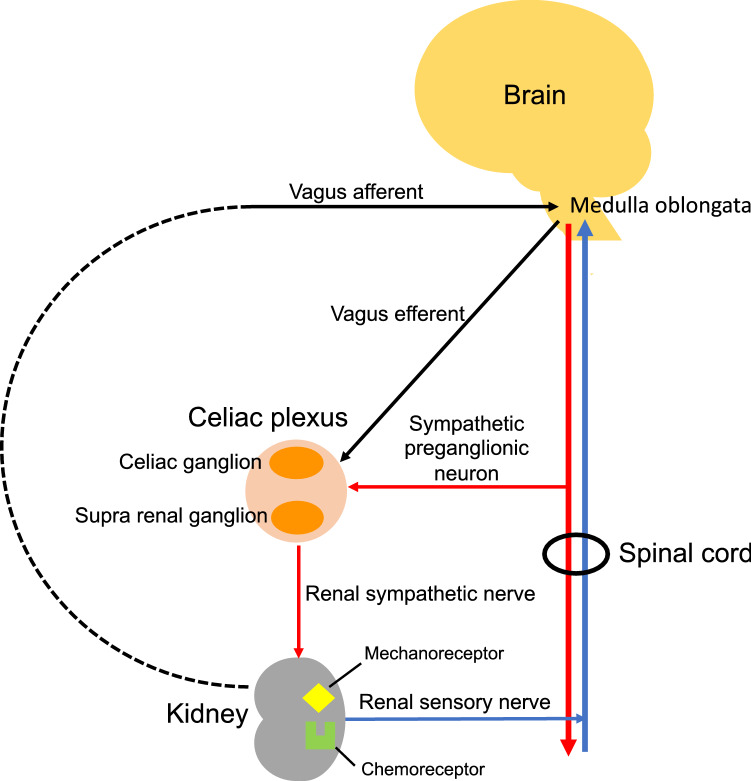
Kidney nervous system. The kidney is mainly innervated by efferent sympathetic nerve fibers that accompany the blood vessels. Sympathetic preganglionic neurons reside in the intermediolateral cell column of the spinal cord synapse with the renal sympathetic nerve in the celiac plexus. Sensory afferent nerves having their cell body in the dorsal root ganglia synapse with neurons in the dorsal horn of the spinal cord transduce information to the medulla oblongata in the brain. Most of the vagal efferent branches begin in the dorsal motor nucleus of the medulla oblongata and branch to organs through intramural ganglia including ganglia located in the celiac plexus. The vagal afferent fibers reach mainly the solitary tract nucleus in the medulla oblongata, but it is not clear whether the afferent fibers from the kidney reach it directly. There seems to be no direct vagus innervation of the kidney. Red lines indicate sympathetic efferent fibers and blue lines show sympathetic afferent (sensory) fibers. Black lines show vagus nerve and dotted line indicates nerve fibers that have not been proven to exist. Orange oval indicates ganglion

In addition to the nervous system- and immune system-mediated kidney protection, I believe that the direct regulation of kidney cells by neurons also plays an important role in kidney protection. Therefore, I have summarized the kidney nervous system in Fig. 6. The relationship between the kidney and the nervous system was first reported in an animal renal denervation model in the mid-nineteenth century [26]. In the 1960s, attention was focused on the regulation of renal renin secretion by the nervous system [27]. Although renal innervation was unknown at that time, direct synaptic connections between nerve fibers inside the kidney and tubule epithelial cells were discovered between 1972 and 1973 [28]. Since then, interest in the regulation of renal function by the nervous system has increased. Further studies revealed that external innervation to the kidney included nerve fibers from the peritoneal plexus, superior mesenteric plexus, and the lumbar visceral plexus [29].

The kidney is the intraperitoneal organ that receives the highest innervation next to the adrenal gland and is innervated by efferent sympathetic nerve fibers that accompany the blood vessels [30, 31]. The renal plexus consists of nerve fibers from the peritoneal ganglion, upper and lower renal ganglia, superior mesenteric ganglion, and thoracic visceral nerve; the renal plexus mainly comprises adrenergic neurons with noradrenaline as the primary neurotransmitter [31]. Although adrenergic neurons mainly control vascular smooth muscle cells, the presence of nerve endings has been confirmed in sites other than blood vessels, suggesting direct control of renal tubules by sympathetic nerves. In animals, the presence of neural junctions [32], and direct sympathetic nerve function regulation, have been observed in renal tubule cells [33]. However, no direct neural innervation of renal tubule cells has been observed in humans.

The afferent sensory nerves of the kidney are mainly located around the renal pelvis [34, 35]. Since they contain neural peptides, such as substance P and calcitonin gene-related peptide, the sensory nerves of the kidney also play roles as mechanical and chemical receptors [36] These nerves are activated by an increase in the internal pressure of the renal pelvis and changes in urinary composition [35]. The cell bodies of these sensory nerves are located in the dorsal root ganglion and synapse, with interneurons in the posterior horn of the spinal cord to transmit information to the central nervous system.

In contrast to the abundant sympathetic innervation, the presence of parasympathetic nerves in the kidney is unclear. The presence of nitric oxide synthase, a parasympathetic marker, was confirmed around the human renal artery [37]. ChAT was found in renal tubules of rats by immunostaining with an antibody. Although it has been reported that ChAT is expressed in the collecting duct [38], innervation of the parasympathetic nerve and its significance in the human kidney remain unknown.

## Conclusion, remarks, and future perspectives

Autonomic nerves directly control the immune system and play an important role in kidney disease. Our findings demonstrate that renal damage can be alleviated by directly manipulating activities of the autonomic nervous system such as by VNS, restraint stress, C1 neuron stimulation with optogenetics, and pulsed ultrasound. However, the renal protection mechanism through nerves has not been fully established, and further studies are required to elucidate these mechanisms. Recently, a method for single-cell RNA-seq using the kidney was developed [39] and various studies utilizing single-cell RNA-seq analysis have been reported in the field of kidney research [40–44]. Indeed, we also elucidated the renal protection mechanism mediated by sympathetic nerve stimulation by utilizing single-cell RNA-seq of the kidney very recently [45]. In addition, in vivo biosensors for neurotransmitters, such as ACh [46] and norepinephrine [47], have been developed recently. Using these biosensors, it is possible to evaluate the activity of autonomic nerves in real time, which would further improve our understanding of the renal protection mechanism through nerves. Our laboratory aims to pave the way for the treatment of kidney disease using these cutting-edge methods.
